# Preliminary clinical outcomes of the double-row anchor suture-bridge technique for the fixation of tibial intercondylar eminence fractures in adults: a 12-months minimal follow-up

**DOI:** 10.1186/s12891-021-03948-9

**Published:** 2021-01-13

**Authors:** Yupeng Chu, Ting Hu, Mangmang Chen, Chendi Jiang, Zhuqi Wu, Junwu Shi

**Affiliations:** Department of Orthopaedic Surgery, The Dingli Clinical Institute of Wenzhou Medical University, Wenzhou, 325000 Zhejiang China

**Keywords:** Suture anchors, Arthroscopic, Suture fixation, Tibial

## Abstract

**Background:**

Tibial intercondylar eminence avulsion fractures occur primarily in adolescents and young adults. However, the incidence of such fractures is increasing in adults, concurrent with an increase in sports injuries and traffic accidents. This study describes the fixation-based double-row anchor suture-bridge technique, a novel technique for treating tibial intercondylar eminence fractures in adults; and evaluates its preliminary clinical outcomes.

**Methods:**

A retrospective evaluation of adult patients with tibial intercondylar eminence fractures treated at our institution from June 2016 to June 2018 was conducted. Seven such patients, treated with the anchor suture-bridge technique, were included. All patients were assessed for knee joint range of motion (ROM), Lysholm knee score, International Knee Documentation Committee (IKDC) Subjective Knee Evaluation Form score, Tegner activity score pre-surgery, and the healing of the fracture at 3, 6 and 12 months minimal post-surgery follow-up.

**Results:**

Patients were followed for a mean of 12.43 months (range 9-15 months). By the final follow-up, all fractures had fully healed. The mean Lysholm score improved from 27.86 (range, 2 to 54) pre-surgery to 88.14 (range, 81 to 100) 3 months post-surgery (*p* < 0.05). Similarly, the mean IKDC score improved from 48.86 (range, 43 to 55) to 84.29 (range, 75 to 90) (*P* < 0.05); and the mean Tegner activity score improved from 1.71 (range, 0 to 4) to 3.29 (range, 2 to 4) (*p* < 0.05). Furthermore, knee joint ROM, Lysholm scores, IKDC scores, and Tegner activity scores displayed excellent outcomes at the 6 and 12 months minimal follow-up.

**Conclusion:**

The arthroscopic anchor suture-bridge technique is a valid and secure method for achieving effective fixation of tibial intercondylar eminence fractures in adults.

## Background

Isolated tibial intercondylar eminence fractures in adults are rare, with most fractures more commonly complicated by combined tibial plateau fractures and/or other injuries. However, the incidence of such isolated fractures has gradually increased in recent decades with an increase in sports injuries and traffic accidents [1, 2]. An avulsion fracture of the tibial intercondylar eminence is regarded as a special type of anterior cruciate ligament (ACL) injury, and its treatment depends on the extent of the fracture’s displacement.

According to the modified Meyers-McKeever-Zaricznyj classification [3, 4], tibial intercondylar eminence fractures can be classified into four types: type I without displacement or slight displacement of fracture fragments; type II with 1/3-1/2 displacement of the anterior edge of avulsed fracture fragments, but no obvious displacement of the posterior edge; type III with complete displacement of fracture fragments; and type IV with comminuted avulsion fractures. Correspondingly, the therapeutic approach for each fracture type differs. Type I fractures are treated conservatively by brace fixation at the extension position of the knee joint. Most type II fractures are surgically treated, although this approach is controversial, and type III fractures are predominantly surgically treated [5, 6]. Anatomical reduction and stable internal fixation are essential for the functional recovery of the knee joint regardless of the patients’ age, especially for patients with cartilage, meniscus or collateral ligament injuries [7].

Arthroscopic internal fixation with tibial tunneling is a classic therapeutic approach for tibial intercondylar eminence fractures. With no agreed upon gold standard, current fixation methods include steel wire, sutures, Endobutton plates, and anchors [6–8]. Of these methods, suture fixation has become the standard method of tibial tunnel fixation. Sutures can penetrate the base of the ACL to effectively fix comminuted fractures and small or thin avulsed fracture fragments. Furthermore, earlier issues related to fixation strength have been addressed by the wide application of high-strength sutures. The anchor suture-bridge technique is a novel surgical approach to treating tibial intercondylar eminence fractures. This technique removes the need to establish an intraoperative bone tunnel, thus avoiding the sequelae of local swelling and effusion, and recent reports have demonstrated its clinical utility [9, 10]. In our study, instead of the locked cinch-knot loop seen in Sawyer’s study, external anchors were used to achieve firmer fixation.

While there have been bio-mechanical studies evaluating the advantages and disadvantages of various fixation methods for ACL tibial insertion avulsion fractures [11, 12], few clinical studies have evaluated the efficacy of the suture-bridge technique for the fixation of tibial intercondylar eminence fractures in adults.

We hypothesized that the double-row anchor suture-bridge technique is the ideal treatment method for providing stable fixation and satisfactory radiographic and clinical outcomes after a 12-months minimal follow-up. The purpose of the study was (1) to describe arthroscopic anchor suture-bridge fixation as a novel technique for treating tibial intercondylar eminence fractures in adults; and (2) to analyze the minimal follow-up results of the radiographic and clinical outcomes.

## Methods

### Patients

**Fig. 1:**
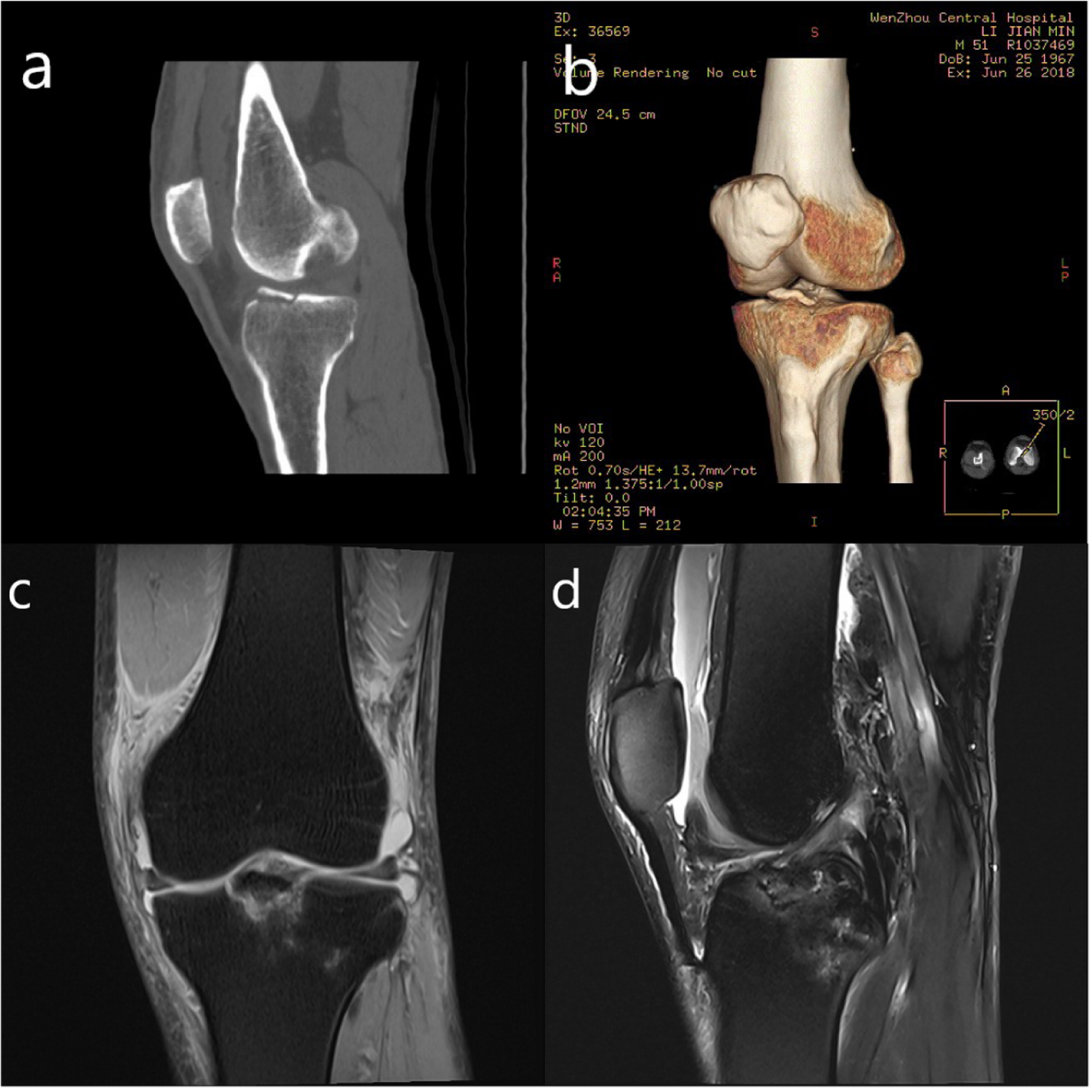
Preoperative images. **a**, **b** Preoperative computed tomography scan showing an example of a type III tibial intercondylar eminence avulsion fractures. **c**, **d** Preoperative MRI result showed a displaced (Type III) avulsion fracture of the tibial intercondylar eminence

A retrospective chart review of adult tibial intercondylar eminence fracture patients treated at our institution between June 2016 and June 2018 was conducted. Seven cases were identified (six males, age range: 28-49 years), all of whom had sport or road traffic related injuries. The inclusion criterion for the study was presence of a displaced ACL avulsion fracture (Meyer and McKeever type II and III); and the exclusion criterion was the presence of Zaricznyj type IV fractures due internal fixation stability. All cases recorded a positive Lachman test during physical examination on admission. Imaging, including X-ray, CT and MRI were performed prior to surgery (Fig. 1) and a meniscus injury was identified in four of the cases. Furthermore, two of the cases were old fractures and five were fresh fractures. Surgery was performed on average 3.8 days (range: 2-6 days) after admission. Patient characteristics are summarized in Table 1.

**Table 1:** The basic characteristics of the patients included

No.	Age	Sex	Injure mechanism	Type	Associated injury	Time from injury to surgery (days)	Operative time(min)	Follow up (months)
001	35	M	Traffic accidents	Type III	Lateral meniscal injury	2	90	15
002	40	M	Sport activities	Type II	None	3	95	12
003	48	F	Sport activities	Type III	Lateral meniscal injury	5	105	12
004	49	M	Traffic accidents	Type II	Lateral meniscal injury	3	120	12
005	44	M	Sport activities	Type III	Lateral meniscal injury	6	100	15
006	37	M	Traffic accidents	Type III	None	4	95	12
007	28	M	Traffic accidents	Type III	None	4	85	12

Patients who met any of the following criteria were excluded: (a) skeletally immature adolescent patients; (b) multiple, open, or pathologic fracture; (c) severe systemic illness (hemophilia, chemotherapy, or a medical contraindication for surgery); and (d) incomplete medical records, or clinical functional assessments. Follow-up indicators were evaluated by the same set of surgeons to reduce artificial error.MRI was repeated 3 days after surgery for each patient and patients were followed-up regularly at 3, 6 and 12 months after surgery. The follow-up examination included the X-ray films of the knee joint, anterior drawer test, Lachmann test, pivot shift test, Lysholm score [13], Tegner score, IKDC rating scale, and the range of motion (ROM) of the knee joint.

### Surgical procedures

All surgical procedures were performed by a single orthopedic surgeon (Junwu Shi). The standard anterolateral and anteromedial approaches to expose the knee joint were made using an arthroscopic system (Smith & Nephew, Andover, MA, USA) with a 30° lens (4 mm). The lumen of the knee joint was examined to check for cartilage and meniscus injuries. One case was complicated by an osteochondral injury to the weight-bearing surface of the medial femoral condyle and underwent microfracture repair [14]. Additionally, two cases were complicated by a longitudinal fissure of the posterior horn of the medial meniscus and underwent a one-stage repair using the FasT-Fix meniscal repair suture system (Smith & Nephew, Andover, MA, USA). Sharp debridement and scar tissue removal around the fracture was carried out, and fracture fragments were reduced using a probe to achieve a congruent articular surface.

Furthermore, in cases of old ACL avulsion fractures, the tibial bed was deepened using a grinding drill. A good reduction was generally obtained. In the case of a poor reduction, free fracture fragments were cleared. Alternatively, the contractural ACL was released moderately (Fig. 3a).

**Fig. 2:**
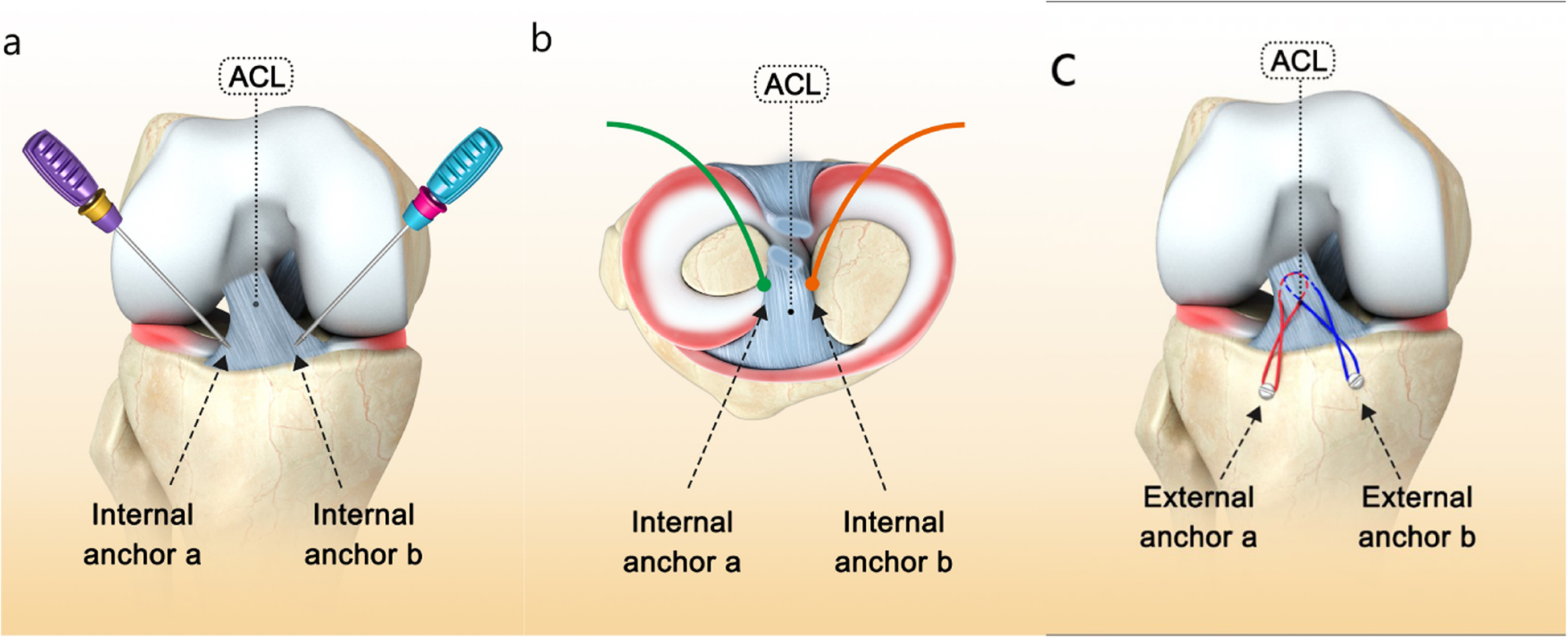
**a** Two internal anchors were placed at the posterior internal and posterior outer edges of the avulsed fracture fragment with an optimal angle of approximately 45° with the tibial plateau; **b** One internal anchor was located at the 2 o’clock point of the bony bed rim and the other was at the 10 o’clock point by taking the arthroscopic field as the reference; **c** Two strands of sutures were mutually crossed and fixed to the anterolateral tibial slope with an external anchor

**Fig. 3:**
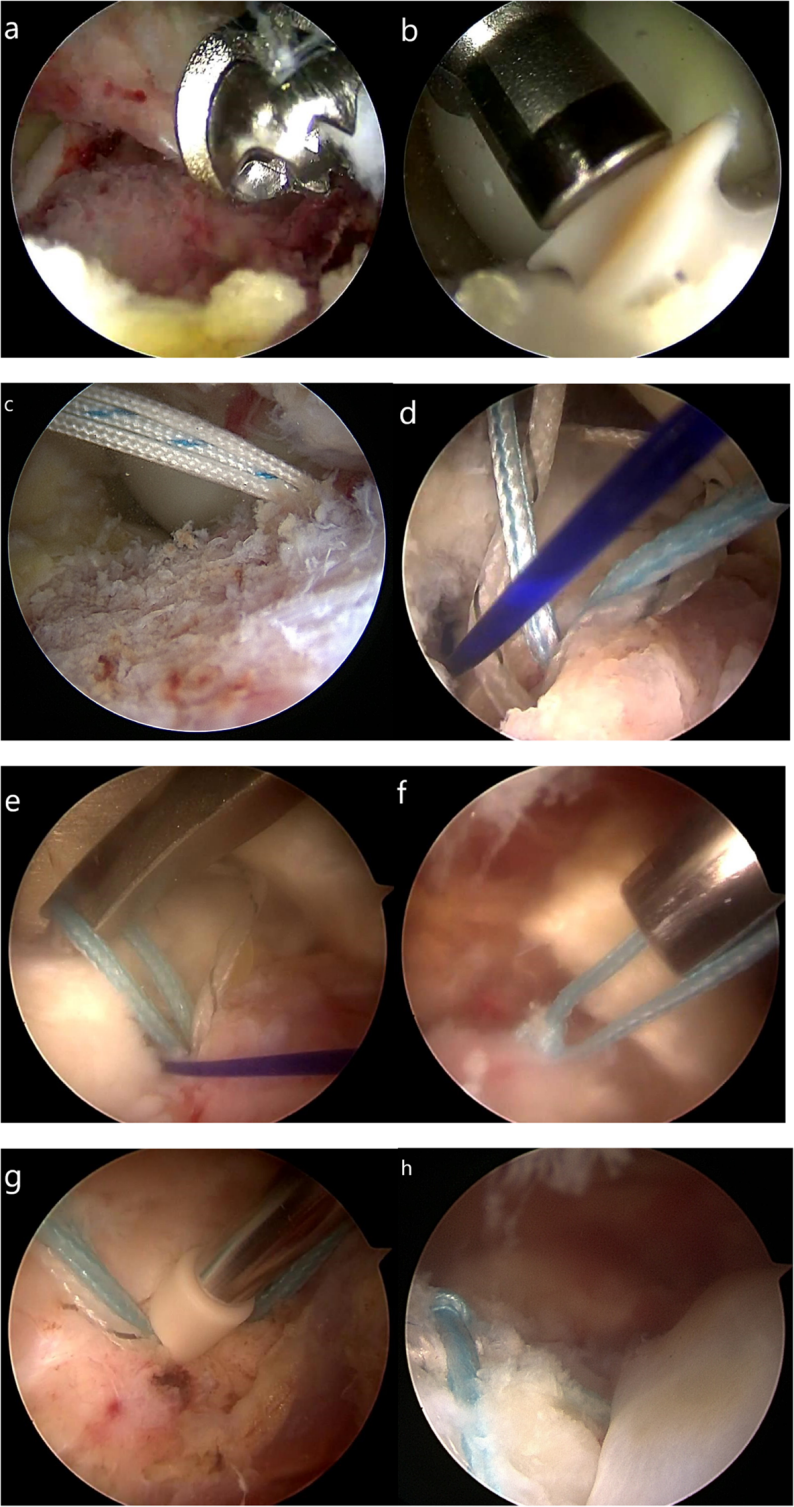
Intraoperative images **a** Displaced tibial eminence fracture under arthroscope. **b**, **c** The internal anchors were placed at the posterior internal and posterior outer edges of the avulsed fracture fragment. **d**, **e** Sutures were passed through the tendon-bone transitional zone adjacent to the fracture fragment. **f** Arthroscopic knot fixation. **g** Two external anchors were implanted. **h** Final suture bridge construct with suture limbs passing over surface of reduced tibial spine fracture

A 15G spinal needle was placed through the anterolateral approach to test the angle of the screw placement, with an optimal angle of approximately 45° with the tibial plateau, as to obtain the best ‘dead angle’ and reduce the risk of pullout (Fig. 3b, c). Following osseous foramen drilling with a mouth-gag, two internal anchors (Arthrex, Naples, USA) were placed at the posterior internal and posterior outer edges of the avulsed fracture fragment (Fig. 2a). Two anchors were sufficient in general, one was located at the 2 o’clock point of the bony bed rim and the other was at the 10 o’clock point by taking the arthroscopic field as the reference (Fig. 2b). After placement of the proximal anchors, eight strands of sutures were smoothly passed through the tendon-bone transitional zone adjacent to the fracture fragment of the ACL insertion using a threader (Fig. 3d, e). Then the sutures were fixed at the tendon-bone junction using a SMC knot secured using a knot-tying device through arthroscopy (Fig. 3f). Next, two strands of sutures were mutually crossed and fixed to the anterolateral tibial slope with an external anchor (Arthrex, Naples, USA). Similarly, two further strands of sutures were mutually crossed and fixed to the anteromedial tibial slope with an external anchor (Arthrex, Naples, USA) (Fig. 2c). An incision reduction of the avulsed fracture fragment was performed with the assistance of a probing hook through the patellar anterior median approach. After adjusting the external anchor position, tightening the suture and fixing the external anchor (Fig. 3g), a satisfactory fracture reduction was confirmed through arthroscopy (Fig. 3h).

### Postoperative management

A cold compress was applied after surgery, routine postoperative antibiotics were administered for 24 h to prevent infection, and detumescence therapy was applied. Vigorous activities were prohibited, and patients were encouraged to engage in active isometric contractions of the quadriceps femoris. Patients were required to wear an adjustable chucker-type knee brace. The ROM was adjusted to 20° 1-week post-surgery, 60° 2- to 3-weeks post-surgery, 90° 4-weeks post-surgery, and 120° 6- to 8-weeks post-surgery and prior to brace removal. After 10- to 12-weeks patients gradually resumed weight-bearing walking, and after 6-months were recovered sufficiently to recommence vigorous activities.

### Statistical analyses

The data of continuous variables were presented as the mean ± standard deviation (SD). All statistical analyses were performed with SPSS 22.0 (SPSS Inc., Chicago, IL, USA). The Wilcoxon rank sum test was used to compare the quantitative data between preoperative and postoperative Lysholm scores, IKDC subjective scores, Tegner activity level scales, and Flexion range of motion. For evaluation of the impact of fracture on the results, the Kruskal–Wallis test was used. A value of *P* < 0.05 was identified as statistically significant.

## Results

**Fig. 4:**
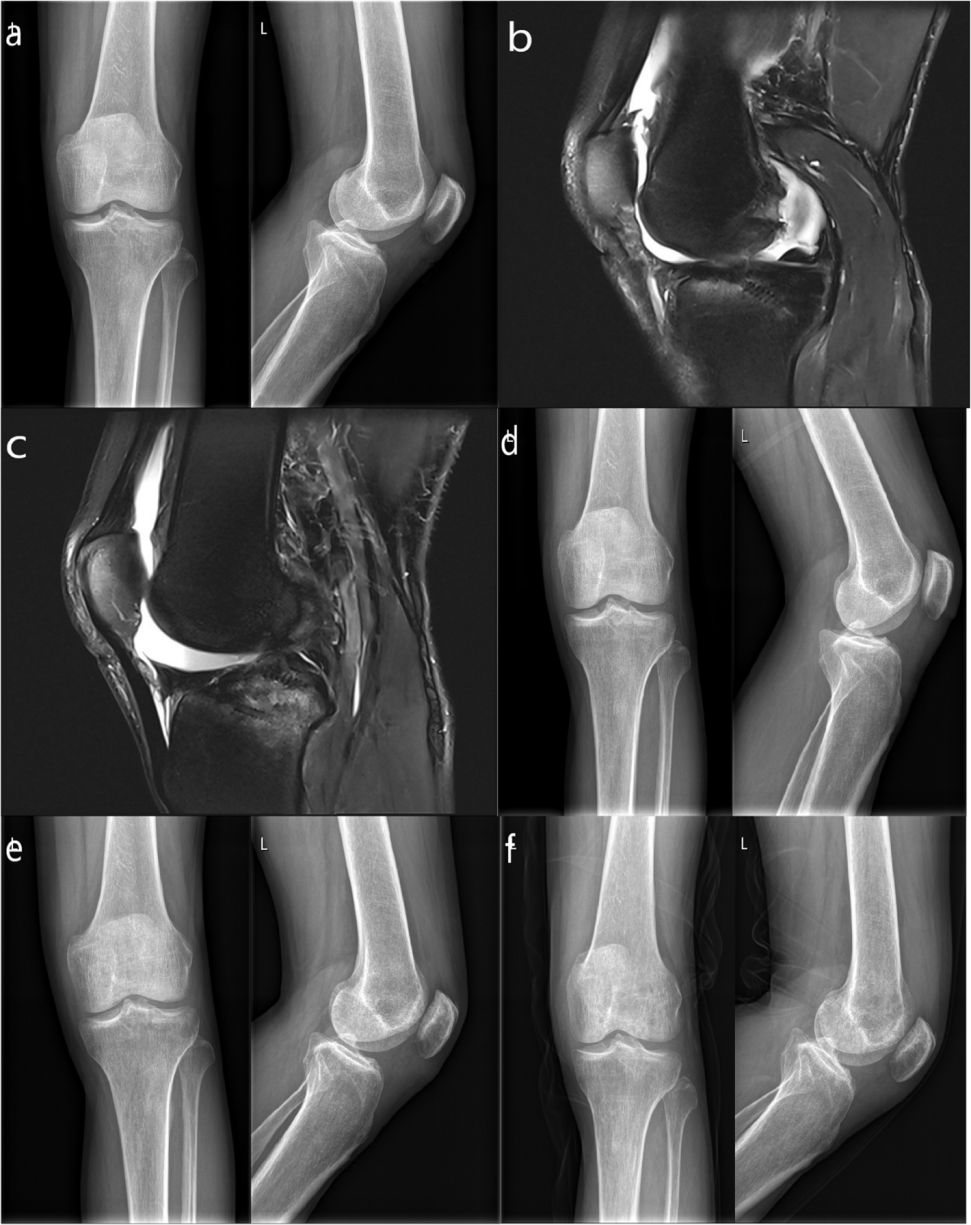
Postoperative images. **a** Postoperative radiographs showed a good reduction of displaced tibial eminence fracture fragment. **b**, **c** MRI image showed the restoration of the displaced fracture fragments and the “Dead Angle” of the suture anchor. **d** The radiograph showed good union of fracture at 3 months after operation. **e** The X-ray image showed excellent union at 6 months after operation. **f** Posterior and lateral radiograph showed a complete bony union at the final following-up

The mean surgical duration was 98.57 min (range, 85-120 min). Patients were followed for a mean of 12.43 months (range 12-15 months). All incisions healed to stage I and no postoperative complications such as infection were observed. A satisfactory fracture reduction was confirmed through X-ray image (Fig. 4a, b, c). X-ray examinations showed good reduction of ACL insertion avulsion fractures 3-months post-surgery (Fig. 4d), and all fractures had healed by 6-months (Fig. 4e).

As summarized in Table 2, at follow-up the mean Lysholm score, IKDC score and Tegner score of the knee joints showed excellent improvement at 6-months and 12-months post-surgery. The mean Lysholm score significantly improved from 27.86 (range, 2 to 54 ) pre-surgery to 88.14 (range, 81 to 100) 3-months post-surgery (*p* < 0.05). Similarly, the mean IKDC and Tegner scores were also significantly improved by 3-months (both *P* values < 0.05). However, there was no significant difference in the mean Lysholm, IKDC or Tegner scores between the post-surgery time points (all *P* values > 0.05). Furthermore, two patients initially developed sustained mild pain in the proximal tibia, however this pain resolved after 3-months (*P* > 0.05, Table 2). The mean knee joint ROM showed significant improvement, increasing from 42.86° (range, 20 to 70°) pre-surgery to 127.14° (range, 110 to 135°) 3-months post-surgery (*P* < 0.05).

**Table 2:** Functional evaluation and stability testings

	Preoperative	Postoperative		
		3 months	6 months	Final
Lyshom score	27.86 ± 24.66	88.14 ± 6.62	94.43 ± 3.41	95.71 ± 3.25
IKDC score	48.86 ± 4.56	84.29 ± 5.35	91.71 ± 3.95	95.43 ± 2.44
Tegner scale	1.71 ± 1.70	3.29 ± 0.76	4.29 ± 0.49	8.14 ± 0.69
Flexion range of motion(degrees)	42.86 ± 22.15	127.14 ± 8.59	129.29 ± 5.35	131.43 ± 2.44

At final follow-up, radiographs showed that all fractures healed during the last follow-up, and no radiographs showed osteolysis or degenerative changes (Fig. 4f). All knees were stable, with negative Lachman examinations and no limitation of motion. All postoperative outcomes showed significant clinical improvement, with measures in the normal or nearly normal range.

## Discussion

Arthroscopic internal fixation through a tibial tunnel is a classic surgical approach for repairing tibial intercondylar eminence fractures. The current preferred approach for type II and III fractures is arthroscopic suture elastic fixation [15]. Both Jang et al. and Boutsiadis et al. demonstrated that ACL avulsion fractures can be fixed by suture via the “three-point method” and “four-point method”, respectively. In both methods, an excellent clinical effect was achieved by replacing one-level fixation with multi-level fixation [6, 8]. Extensive recent developments in the arthroscopic double-row anchor suture-bridge technique for the repair of rotator cuff injuries has resulted in excellent clinical outcomes [16, 17]. Furthermore, this technique has been utilized in the treatment of tibial intercondylar eminence fracture, and reported as having good effect [9, 10]. In Sawyer’s study, a suture shuttle device is used to pass FiberWire sutures, placed in a locked cinch-knot loop, through the anterior ACL adjacent to the fracture fragment. In our study, the application of external anchors was shown to be convenient for operation and could achieve firmer fixation. At present, there are few studies regarding the anchor suture-bridge technique for the treatment of tibial intercondylar eminence fractures in adult. This study was conducted to assess the preliminary clinical outcomes of this technique to guide its future application in clinical practice.

In the present study, all patients displayed significant post-surgery clinical and radiological improvements . This supports the proposition that the anchor suture-bridge technique is an effective fixation method for the repair of adult tibial intercondylar eminence fractures. This conclusion is supported by a systematic retrospective analysis by Osti et al. which compared different internal fixation methods such as steel wire, screw, non-absorbable suture, and anchor. While the different methods all produced good clinical and imaging results, suture techniques could reduce the degree of iatrogenic trauma owing to the absence of a secondary operation to remove internal fixation [12]. In this study, the suture-bridge technique resulted in reasonably early stability and in the ability to commence functional exercises. A good mid/long-term effect was also achieved, with no difference in function between 6 months and 12-months post-surgery.

Arthroscopic trans-bone tunnel fixation is the most commonly used procedure for ACL fractures and avulsion fractures of the tibial insertion. To date, several fixation methods are commonly used. For example, Schneppendahl et al. reported a good fixation effect using three absorbable sutures in the treatment of ACL avulsion fractures in children [18]. Similarly, Liao et al. reported no difference between absorbable and non-absorbable sutures for the repair of tibial intercondylar eminence fractures [19]. Sekiya et al. showed that an Endobutton plate was a simple, effective and firm internal fixation method for treating a tibial intercondylar eminence fracture through a proximal tibial bone tunnel [20]. Interestingly, Hapa et al. compared hollow nail, high-strength suture, Endobutton plate and anchor methods in repairing tibial intercondylar eminence fractures with respect to their biomechanics and suggested that suture fixation could provide a firm fixation despite its disadvantage relating to a weaker fixation force compared to an Endobutton plate [21]. The tibial intercondylar eminence is the attachment area of the ACL insertion and includes the footprint. This type of fracture is characterized by an avulsion fracture, which is categorized as an ACL injury. In a description of the surgical technique by Sawyer et al. [11], an arthroscopic suture-bridge was applied for the treatment of a tibial insertion avulsion fracture of the cruciate ligament. By adopting this method, the bone mass and tibial plateau could provide a greater contact area to form the plane fixation, which was more reliable than other techniques, and achieved a good clinical outcome.

In practice, the insertion point of the internal anchor should be placed as far as possible under the medial and lateral tibial intercondylar cartilage at a 45° angle to the tibial cartilage surface as to obtain the optimum dead angle resulting in the best stability. Highlighting the importance of establishing this optimal stability, Li et al compared the mechanical stability of screw, absorbable suture and anchor suture-bridge methods for the treatment of ACL avulsion fractures, which demonstrated that the anchor suture-bridge technique exhibited superior biomechanical properties [22]. However, it should be acknowledged that the suture-bridge technique is relatively complicated compared to other methods. There is a need for anchor implantation and repeated threading through the patellar anterior median incision, resulting in a prolonged surgery. In this study, the average surgical time was 98.57 min. Post-surgical MRIs showed that all suture anchors were settled at the optimal dead angle and the displaced tibial eminence fracture fragments were reduced to a good position (Fig. 4b, c). At final follow-up, posterior and lateral X-rays showed complete bony union (Fig. 4f).

At present, numerous procedures for the repair of ACL avulsion fractures have been developed focusing on addressing anatomical reduction of fracture fragments and recovery of cruciate ligament tension [23]. However, several issues remained to be addressed, such as knee extension dysfunction and ACL relaxation. A potential reason for these continuing issues may be the practice of selecting the fixation method primarily based on the classification of the fracture, given that the current classification system does not reflect the degree of soft tissue injury [24]. As demonstrated by Xu et al., mid-term follow-up of displaced tibial intercondylar eminence fractures repaired using the anchor suture-bridge technique resulted in significant post-surgical outcomes (as measured by Lysholm, IKDC and KT-1000 scores), suggesting that the anchor suture-bridge technique has obvious advantages in the treatment of tibial intercondylar eminence fractures [25]. Furthermore, Verdano et al. also showed viable repair of tibial intercondylar eminence fractures by arthroscopic suture fixation [26].

This study has some limitations. Firstly, as a retrospective analysis, the present study provides only a limited level of evidence regarding clinical efficacy. Secondly, the sample size was small, and the timespan of case selection was wide, which may have resulted in selection bias. Thirdly, the study did not include cases of type IV comminuted fractures and small avulsed bone fragments, limiting its generalizability to clinical practice.

## Conclusions

The arthroscopic double-row anchor suture-bridge technique can achieve effective fixation of tibial intercondylar eminence fractures in adults. This suture-bridge technique produces rapid healing and good functional stability, allowing for the resumption of functional exercise. However, the technique is slightly more complicated than alternative approaches, and requires a high degree of skill and patience for knee arthroscopy.

## Data Availability

Not applicable. This study was only the primary research, and further study has been in progress.

## Abbreviations

ACL: Anterior cruciate ligament
CT: Computerized tomography
IKDC: International Knee Documentation Committee
MRI: Magnetic resonance imaging
ROM: Range of motion
